# Omission

**Published:** 1881-08

**Authors:** 


*Omission.*—The report of the proceedings of the Maryland and District Columbia Dental Association, as printed in this Journal, had omitted from it the names of the "Committee on Scholarship." The committee is as follows:

Dr. J. Curtiss Smithe, D. C., Dr. M. W. Foster, Baltimore, Dr. H. C. Thompson, D. C.

